# HK3 overexpression associated with epithelial-mesenchymal transition in colorectal cancer

**DOI:** 10.1186/s12864-018-4477-4

**Published:** 2018-02-09

**Authors:** Elena A. Pudova, Anna V. Kudryavtseva, Maria S. Fedorova, Andrew R. Zaretsky, Dmitry S. Shcherbo, Elena N. Lukyanova, Anatoly Y. Popov, Asiya F. Sadritdinova, Ivan S. Abramov, Sergey L. Kharitonov, George S. Krasnov, Kseniya M. Klimina, Nadezhda V. Koroban, Nadezhda N. Volchenko, Kirill M. Nyushko, Nataliya V. Melnikova, Maria A. Chernichenko, Dmitry V. Sidorov, Boris Y. Alekseev, Marina V. Kiseleva, Andrey D. Kaprin, Alexey A. Dmitriev, Anastasiya V. Snezhkina

**Affiliations:** 1 Engelhardt Institute of Molecular Biology, Russian Academy of Sciences, Moscow, Russia; 2 National Medical Research Radiological Center, Ministry of Health of the Russian Federation, Moscow, Russia; 3 Pirogov Russian National Research Medical University, Moscow, Russia; 4 Vavilov Institute of General Genetics, Russian Academy of Sciences Moscow, Moscow, Russia; 5 Pletnev State Hospital, Moscow, Russia

**Keywords:** Warburg effect, Hexokinase, Glycolysis, Colorectal cancer, Epithelial-mesenchymal transition

## Abstract

**Background:**

Colorectal cancer (CRC) is a common cancer worldwide. The main cause of death in CRC includes tumor progression and metastasis. At molecular level, these processes may be triggered by epithelial-mesenchymal transition (EMT) and necessitates specific alterations in cell metabolism. Although several EMT-related metabolic changes have been described in CRC, the mechanism is still poorly understood.

**Results:**

Using CrossHub software, we analyzed RNA-Seq expression profile data of CRC derived from The Cancer Genome Atlas (TCGA) project. Correlation analysis between the change in the expression of genes involved in glycolysis and EMT was performed. We obtained the set of genes with significant correlation coefficients, which included 21 EMT-related genes and a single glycolytic gene, *HK3*. The mRNA level of these genes was measured in 78 paired colorectal cancer samples by quantitative polymerase chain reaction (qPCR). Upregulation of *HK3* and deregulation of 11 genes (*COL1A1, TWIST1, NFATC1, GLIPR2, SFPR1, FLNA, GREM1, SFRP2, ZEB2, SPP1,* and *RARRES1*) involved in EMT were found. The results of correlation study showed that the expression of *HK3* demonstrated a strong correlation with 7 of the 21 examined genes (*ZEB2, GREM1, TGFB3, TGFB1, SNAI2, TWIST1,* and *COL1A1*) in CRC.

**Conclusions:**

Upregulation of *HK3* is associated with EMT in CRC and may be a crucial metabolic adaptation for rapid proliferation, survival, and metastases of CRC cells.

**Electronic supplementary material:**

The online version of this article (10.1186/s12864-018-4477-4) contains supplementary material, which is available to authorized users.

## Background

In 2012, an estimated 1.36 million people were diagnosed with colorectal cancer (CRC), including 746,300 men and 614,300 women. The mortality rate of CRC is reported to be approximately half of its global incidence. In the same year, 694,000 people died from CRC [1], making it the third most common cancer and fourth most common cause of death worldwide [2].

The stage of CRC plays a significant role in survival of patients. More than 60% of patients are diagnosed at late stage of CRC (III-IV) and approximately 25% of them display metastatic disease [3, 4]. Development of new drugs and targeted therapies has dramatically improved the survival of these patients over the last decade [5]. However, the survival outcome in patients with metastatic or stage IV CRC still remains poor [6, 7]. For instance, the 5-year relative survival rate for patients with metastatic CRC is about 11%, while that for patients with stage III CRC ranges from 53% to 89% (https://www.cancer.org). CRC is characterized by severe genetic alterations, including microsatellite instability (MSI), epigenetic changes such as DNA methylation (CpG Island Methylator Phenotype [CIMP]), and chromosomal instability (CIN), which are associated with the development of the disease and its aggressive behavior [8, 9]. Thus, identification of the distinct molecular genetic changes related to CRC progression and metastasis is extremely important.

A number of studies have demonstrated that epithelial-mesenchymal transition (EMT) is involved in CRC carcinogenesis and metastasis [10, 11]. EMT is the conversion of epithelial cells into cells with a mesenchymal phenotype, which is accompanied by a decrease in cell-cell cohesion and fundamental reorganization of the cytoskeleton. Consequences include enhanced migratory as well as invasive capacity and resistance to apoptosis [12, 13]. Based on the biological context, three types of EMT have been described as follows: type I observed in embryogenesis [14], type II involved in tissue repair and fibrosis [15], and type III seen in metastatic transformation of cancer cells [16]. A hallmark feature of EMT is the downregulation of E-cadherin, a cell-cell adhesion molecule localized on the surface of epithelial cells [17]. E-cadherin is a well-known tumor suppressor protein and loss of its expression in tumor cells is associated with increased tumor invasiveness and metastasis [18, 19]. In CRC, EMT is characterized by decreased E-cadherin expression and nuclear accumulation of β-catenin, which is an essential protein for correct positioning and function of one [20, 21]. Loss of epithelial and gain of dedifferentiated mesenchyme-like phenotype enable CRC cells to develop invasive and metastatic growth characteristics [22]. The switch from cytoplasmic to nuclear accumulation of β-catenin is a central mechanism related to EMT in CRC [21]. This process may be mediated by the loss-of-function mutations in the adenomatous polyposis coli (APC) tumor suppressor gene or mutations that result in β-catenin stabilization [23]. In the nucleus, β-catenin associates with DNA-binding proteins of the T-cell factor/lymphoid enhancer factor (TCF/Lef) family, leading to a constitutive activation of Wnt/β-catenin signaling target genes, *c-Myc* [24] and *CCND1* [25]. Moreover, β-catenin triggers the expression of other genes such as *MMP7* [26, 27], *FN* [28], *CD44* [29], and *UPAR* [30] involved in invasive cell growth. Except for this mechanism, there are at least ten known signaling pathways and many molecules associated with EMT in CRC [31].

Metabolic reprogramming characterized by upregulated glycolysis is another crucial feature of cancer. It provides cancer cells with energy and metabolites essential for active cell division, large-scale biosynthesis, invasion, and metastasis [32–34]. Increased glycolysis has been suggested as an essential component of the malignant phenotype and hallmark of invasive cancers [35]. In CRC, redirection of glucose metabolism is promoted by deregulation of key components involved in glucose transport (GLUT-1) [36], metabolism (hexokinases [HK1, HK2], pyruvate kinase M2 [PKM2], lactate dehydrogenase A [LDHA], aldolase A [ALDOA], etc.) [37–39], as well as metabolic regulation [40–42]. In addition, variation in the representation of alternative spliced transcripts related to energy metabolism seems to be associated with the prevalence of aerobic glycolysis in cancer cells [43, 44]. EMT in tumors requires the distinct metabolic adaptations for survival, rapid proliferation, and metastasis. However, metabolic changes observed upon EMT induction are uncommon for various cancer types [45–47]. Recent studies showed that the glycolytic enzymes aldolase B (ALDOB), PKM2, and glyceraldehyde-3-phosphate dehydrogenase (GAPDH) play a role in EMT and metastasis in CRC [48, 49].

In this work, using CrossHub software we analyzed RNA-Seq data from The Cancer Genome Atlas (TCGA) project to estimate the association between the expression of genes participating in EMT and glycolysis in CRC. Hexokinase 3 (HK3) was found to exhibit significant correlation with a set of genes involved in EMT. Thus, we focused on the investigation of *HK3* gene expression in CRC samples and its association with EMT.

## Methods

### Bioinformatic analysis

Using CrossHub software [50], we have analyzed The Cancer Genome Atlas (TCGA, https://cancergenome.nih.gov) project RNA-Seq dataset derived from CRC. The dataset contained 287 tumor samples including 26 paired ones. The selection of genes participating in glycolysis and EMT was performed using Gene Ontology database and “glycolytic process” and “epithelial to mesenchymal transition” as keywords. A set of 132 genes was selected for further analysis.

The correlation analysis between the expressions of target genes was performed using Spearman’s rank correlation coefficients (*r*_*s*_). We carried out two tests: one for paired samples and other for pool of tumor samples. Spearman’s rank correlation coefficients were calculated between (1) fold change values, the ratio of CPM (counts per million) in tumor sample to CPM in conditional normal tissue, (paired samples) and (2) CPM values (pool of tumor samples). Before calculating correlation coefficients, dropping points (no more than 5%) were eliminated using approximate generalized linear model. We focused on the genes with concordant results in paired and pooled tests.

### Tissue samples

In total, 78 paired CRC samples, including tumor and adjacent morphologically normal tissues (conditional normal tissue), were collected from patients in Herzen Moscow Cancer Research Institute - branch of National Medical Research Radiological Center, Ministry of Health of Russian Federation (Moscow, Russia). The sample information is presented in Table 1. Samples were obtained after surgical resection prior to radiation or chemotherapy and were stored in liquid nitrogen. The diagnosis was verified by histopathology, and only samples with 70% or more tumor cells were used in the study. The morphological classification of the tumor was performed according to the WHO Classification of Tumours of the Digestive System (WHO/IARC Classification of Tumours, 4th Edition, 2010) and verified according to the American Joint Committee on Cancer (AJCC) staging system (AJCC Cancer Staging Manual, 8th Edition, 2017). Samples were collected in accordance with the guidelines issued by The Ethics committee of Herzen Moscow Cancer Research Institute. Written informed consent was obtained from all patients.

**Table 1:** Clinicopathologic characteristics of the tumors

Characteristic	Total, n
Gender	
Male	46
Female	32
Age (years)	
≤ 60	26
> 60	52
Clinical stage	
I	2
II	28
III	31
IV	17
Distant metastases (Stage IV)	
Negative	4
Positive	13

### RNA isolation and reverse transcription

Nitrogen-frozen tissues were homogenized using a Mikro-Dismembrator S (Sartorius, Germany). Total RNA was isolated using the RNeasy Mini Kit (Qiagen, Germany) according to manufacturer’s instructions. Purified RNA was quantified using NanoDrop 1000 (NanoDrop Technologies Inc., USA) and the RNA quality measured with the RNA Integrity Number (RIN) method on Agilent RNA Bioanalyzer 2100 (Agilent Technologies, USA). All RNA samples were treated with RNase-free DNase I (Thermo Fisher Scientific, USA). Reverse transcription was performed from 1 μg of total RNA using M-MuLV reverse transcriptase (Thermo Fisher Scientific) and random hexamer primers (Evrogen, Russia). Negative control samples were included in each set of reactions. Reactions were heated at 70 °C for 5 min to melt the secondary structure of mRNA, followed by immediate cooling on ice. Samples were further incubated at 25 °C for 5 min, followed by treatment with M-MuLV reverse transcriptase at 25 °C for 10 min. Then the reaction tubes were incubated at 42 °C for 1 h. Following the incubation, samples were diluted to 48 μL in nuclease-free water and stored at − 20 °C.

### Quantitative polymerase chain reaction (qPCR)

Quantitative polymerase chain reaction was performed using commercial primer-probe sets for target genes (*HK3*: Hs01092850_m1, *VASN*: Hs01936449_s1, *GREM1*: Hs01879841_s1, *TGFB3*: Hs01086000_m1, *TGFB1*: Hs00998133_m1, *LOXL3*: Hs01046941_g1, *HGF*: Hs00300159_m1, *SNAI2*: Hs00161904_m1, *FAM101B*: Hs00823804_m1, *SFRP2*: Hs00293258_m1, *FLNA*: Hs00924645_m1, *WWTR1*: Hs00210007_m1, *SFRP1*: Hs00610060_m1, *GLIPR2*: Hs01555479_m1, *NFATC1*: Hs00542675_m1, *TWIST1*: Hs01675818_s1, *COL1A1*: Hs00164004_m1, *ZEB2*: Hs00207691_m1, *VIM*: Hs00958111_m1, *TP53*: Hs01034249_m1, *SPP1*: Hs00959010_m1, *RARRES1*: Hs00894859_m1) from TaqMan Gene Expression Assays (Thermo Fisher Scientific). Primers and probes for reference genes, *GUSB* and *RPN1*, were designed earlier [51]. The reactions were carried out on AB 7500 Real-Time PCR System (Thermo Fisher Scientific) as previously described [52]. Each reaction was repeated three times.

QPCR data were analyzed using relative quantification or ΔΔCt-method as previously described [44, 52]. Relative mRNA level of the genes was calculated using ATG program compatible with relative quantification software (Thermo Fisher Scientific) [52]. Given the variability of mRNA level of the reference gene, an mRNA level change of at least two-fold was considered as significant.

### Statistical analysis

Statistical analysis was performed using SPSS 10 software (SPSS Inc., USA). The Wilcoxon/Mann-Whitney and Kruskal-Wallis tests were applied to analyze differences in mRNA expression of target genes in CRC samples. Spearman’s rank correlation coefficient (*r*_*s*_) was used for revealing correlations. A value of *p* ≤ 0.05 was considered statistically significant.

## Results

### Preliminary correlation analysis of gene expression based on TCGA project data

We identified a number of genes that showed high correlations between each other in CRC with the false discovery rate (FDR) ≤ 0.05. In the heat maps, these genes formed a significant correlation module (Additional file 1: Table S1 and Additional file 2: Table S2). Only one glycolytic gene, *HK3,* was presented in this module. A significantly strong correlation was observed between *HK3* gene and 16 EMT-related genes (*VASN, GREM1, TGFB3, TGFB1, LOXL3, HGF, SNAI2, FAM101B, SFRP2, FLNA, WWTR1, SFRP1, GLIPR2, NFATC1, TWIST1,* and *COL1A1*), both in paired as well as pool of tumor samples. In addition, literature data analysis revealed several genes that are involved in EMT process. Preliminary calculation of correlation coefficients between the expression of these genes and *HK3* was carried out using CrossHub software and RNA-Seq data from TCGA project as described above. The results obtained are shown in Additional file 3: Table S3 (available only for 26 paired CRC samples). A significant correlation was reported between the change in expression of *HK3* gene and five additional genes (*ZEB2, VIM, TP53, SPP1,* and *RARRES1*) involved in EMT. Thus, *HK3* and a set of 21 EMT-related genes were selected for further assessment by qPCR.

### Upregulation of HK3 gene expression in CRC

**Fig. 1:**
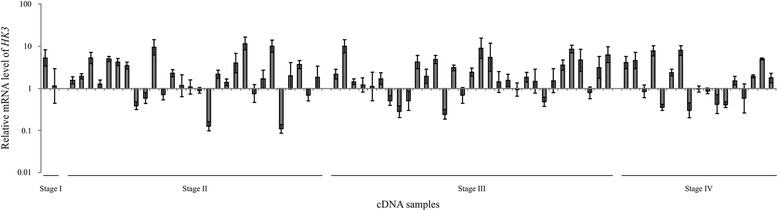
Relative mRNA level of *HK3* gene in colorectal cancer (qPCR data). The mRNA level of *HK3* was normalized by those of two reference genes, *GUSB* and *RPN1*. Data were presented according to CRC progression stage

Using qPCR, we quantified the expression of *HK3* gene in 78 paired CRC samples. *HK3* mRNA level was upregulated from 2 to 12-fold in 41% (32/78, *p* < 0.01) CRC samples as compared with that in conditional normal tissue (Fig. 1). Over two-fold decrease in the expression of *HK3* gene was determined in 14% (11/78) of cases. The mean value of relative mRNA level was 1.7. *HK3* overexpression was not correlated with CRC progression stage.

In addition, we verified the expression of all selected EMT-related genes using qPCR. Deregulation in the expression of 11 genes (*COL1A1, TWIST1, NFATC1, GLIPR2, SFPR1, FLNA, GREM1, SFRP2, ZEB2, SPP1,* and *RARRES1*) was detected in CRC (Table 2). A significant increase (*p* < 0.05) in the mRNA level of *COL1A1, TWIST1, CREM1*, and *SPP1* genes was observed in most examined samples. Moreover, *NFATC1, GLIPR2, SFPR1, FLNA, SFRP2, ZEB2,* and *RARRES1* genes were downregulated by over two-fold (*p* < 0.05) in more than 40% of CRC cases.

**Table 2:** Frequency and mRNA level changes of eleven EMT-related genes in CRC

Gene	Frequency of mRNA level changes, %		Median of mRNA level changes, n-fold
	**↑**	**↓**	
*COL1A1*	79.5 (62/78)	5 (4/78)	5.5 ↑
*TWIST1*	67 (52/78)	4 (3/78)	2.9 ↑
*NFATC1*	9 (7/78)	50 (39/78)	1.6 ↓
*GLIPR2*	0 (0/78)	74 (58/78)	3.1 ↓
*SFPR1*	4 (3/78)	82 (64/78)	15.9 ↓
*FLNA*	15 (12/78)	44 (34/78)	1.8 ↓
*GREM1*	53 (41/78)	20.5 (16/78)	1.5 ↑
*SFRP2*	23 (18/78)	46 (36/78)	2.2 ↓
*ZEB2*	3 (2/78)	55 (43/78)	2.1 ↓
*SPP1*	76 (59/78)	5 (4/78)	7.9 ↑
*RARRES1*	9 (7/78)	63 (49/78)	2.2 ↓

*Note:* QPCR data. ↓/↑: mRNA level decrease/increase

### Correlation study between HK3 and EMT-related genes in CRC

In order to determine the correlation between *HK3* and EMT-related genes, Spearman’s rank correlation coefficients were calculated. The results observed are shown in Table 3. We found a significantly strong correlation (> 0.5 or < − 0.5, *p* < 0.05) between *HK3* mRNA level and expression of seven of 21 examined genes (*ZEB2, GREM1, TGFB3, TGFB1, SNAI2, TWIST1,* and *COL1A1*) in CRC. Generally, *HK3* gene expression was positively correlated with almost all tested genes at moderate or weak levels. A negative correlation was detected between *HK3* and *TP53* genes only.

**Table 3:** Results of Spearman’s correlation analysis between the mRNA levels of *HK3* and EMT-related genes

Gene	Correlation coefficient, *r*_*s*_
*ZEB2*	0.62*
*VIM*	0.49*
*VASN*	0.33*
*GREM1*	0.51*
*LOXL3*	0.39*
*HGF*	0.20
*TP53*	−0.47*
*TGFB3*	0.57*
*TGFB1*	0.59*
*SNAI2*	0.58*
*SPP1*	0.44*
*RARRES1*	0.35*
*SFRP2*	0.39*
*FLNA*	0.12
*WWTR1*	0.15
*SFRP1*	0.29
*GLIPR2*	0.23
*NFATC1*	0.42*
*TWIST1*	0.52*
*COL1A1*	0.53*
*FAM101B*	0.48*

*- *p* < 0.05

## Discussion

In the 1920s, Otto Warburg and co-workers observed the altered energy metabolism in proliferating tumor cells. These cells were shown to exhibit increased glycolysis with lactate secretion even in the presence of oxygen — a phenomenon known as the “Warburg effect” [53]. Increased glucose metabolism, now considered as a hallmark of cancer, is thought to be essential for tumor cell growth, survival, proliferation, and long-term maintenance [54]. The first step of glycolysis is catalyzed by hexokinases (HKs). There are four HK isoenzymes encoded by separate genes*, HK1, HK2, HK3*, and *HK4* (or *GCK*) [55]. HKs phosphorylate glucose to glucose 6-phosphate using ATP molecule as the phosphoryl donor [56]. HKs are the first rate-limiting enzymes of glycolysis. Alterations in their expression promote changes in glucose flux. Overexpression of *HK1* and *HK2* genes was reported in many tumors including colorectal, prostate, breast, lung, gastrointestinal, and pancreatic cancers [39, 57–61]. In particular, HK2 expression was suggested to be responsible for accelerated glycolysis in cancer cells [62]. Nevertheless, activities of both HK1 and HK2 are essential for their survival [63]. Association of HK4 expression with tumorigenesis is still unclear. However, polymorphisms in HK4 and HK2 affect the risk and clinical outcome in pancreatic cancer [64, 65]. A recent study reported functional role of HK3 in acute promyelocytic leukemia [66, 67]. No other studies have described the role of HK3 in human cancers. This is the first study to report *HK3* upregulation in CRC and its potential association with EMT.

The loss of E-cadherin expression during EMT is a major event in cancer. Downregulation of E-cadherin is mediated by a series of transcription repressors, including proteins of the SNAIL superfamily (SNAI1, SNAI2, and SNAI3) and ZEB family (ZEB1 and ZEB2), forkhead-box protein FOXC2, factors E47 and KLF8, and TWIST bHLH proteins (TWIST1 and TWIST2) [68]. Upregulation of major EMT transcription factors such as TWIST1, ZEB1, ZEB2, SNAI1, and SNAI2 has been observed in many tumors [69–77]. We showed an increased expression of *TWIST1* gene in CRC, as reported in a previous study [78]. Furthermore, overexpression of TWIST1 was demonstrated to promote migration and invasion of CRC cells and induce EMT. Although no significant upregulation in *SNAI2* mRNA level was observed in this work, SNAI2 protein expression was earlier shown to be upregulated in colorectal tumors [77]. Shioiri and co-authors demonstrated the localization of overexpressed SNAI2 protein in the cytoplasm, wherein its gene-repressive function is absent [79]. We also reported downregulation of *ZEB2* gene in more than half of CRC samples, while its overexpression was observed at the invasion front of CRC in another study [80]. However, *ZEB2* protein promotes EMT progression through direct suppression of the transcription of genes involved in epithelial dedifferentiation [81, 82]. Thus, *ZEB2* overexpression may not be generally observed after EMT and may not always correlate with the mesenchymal phenotype [83]. In addition, we found significant positive correlations between the expression of *HK3* and *TWIST1*, *SNAI2*, and *ZEB2* genes in CRC. The association of *HK3* with important EMT inducers may indicate its involvement in EMT in colorectal cancer.

The genes *TGFB1* and *TGFB3* encode signaling proteins, transforming growth factor-beta 1 and 3 (TGFβ1 and TGFβ3). These are secreted ligands of specific TGFβ membrane receptors that play an essential role during differentiation, proliferation, and embryonic development in normal tissues. In general, the binding of the ligand to its receptor initiates phosphorylation of proteins belonging to the SMAD family. Activated SMAD proteins assemble into complexes with transcription factors to directly regulate gene expression [84]. Many studies have demonstrated the involvement of TGFβs in EMT during normal development and pathological processes [85–88]. Alterations in the components of TGFβ pathway are often observed during CRC progression. Approximately 40–50% of all CRC cases display mutational inactivation of this signaling pathway [89, 90]. Although TGFβ pathway exhibits a tumor-suppressive role, increased level of TGFβ1 in plasma of patients with CRC was associated with the development of metastasis [91]. Moreover, elevated TGFβ1 level has been reported to increase the metastasis capability of CRC cells and inhibition of TGFβ receptor 1 resulted in metastasis formation in animal model [92]. No significant change in the expression of *TGFB1* and *TGFB3* genes in CRC was observed in our study. However, these genes demonstrated a strong positive correlation with *HK3* mRNA level.

We observed a significant upregulation in *GREM1* and *COL1A1* gene expression in CRC. A positive correlation was observed between these genes and *HK3*. *GREM1* gene encodes a member of the BMP antagonist family and is known as a mediator of EMT in non-cancerous pathologies and cancer [93–96]. GREM1 was shown to be involved in the migration of CRC cells in vitro and in silico [97]. However, a few studies have demonstrated an association between GREM1 expression and overall survival of CRC patients [98, 99]. *COL1A1* gene encodes for alpha 1 type I collagen, a target protein for TGFβ1 transcription factor. Type I collagen is associated with EMT in normal and cancer tissues [100–102]. It may downregulate E-cadherin and β-catenin and promote EMT-like phenotype in CRC cells [103]. In the study by Zou and co-workers, overexpression of *COL1A1* gene was observed in tissues and serum samples of patients with CRC. The serum level of COL1A1 correlated with the staging and poor survival rate of CRC [104].

It should to be noted that *TP53* gene encodes a tumor suppressor protein, p53. One of the most important functions of p53 is the activation of apoptosis. *TP53* is frequently mutated in human cancers, and over 50% of all tumors display somatic mutations in *TP53* gene [105]. Loss of p53 activity is also associated with EMT phenotype. It has been shown that downregulation of p53 promotes proliferation, EMT-mediated migration, and invasion of CRC cells [106]. In addition, p53 was recently found to be involved in cancer cell metabolism [107, 108]. p53 displays an ability to suppress glycolysis and stimulate oxidative phosphorylation through transcriptional regulation of several glycolysis-related genes [109–111]. We observed a significant negative correlation between mRNA levels of *HK3* and *TP53* genes. Thus, *HK3* gene could be a potential p53 target and suppression of p53 may not only contribute to increased glycolysis but also provide some advantages for EMT in CRC cells. It is interesting that the upregulation of *HK3* expression was observed in breast cancer samples with *TP53* mutations, as per TCGA data [112]. In this study, CRC samples were not tested for *TP53* mutations, but could also be positive for ones.

## Conclusions

We showed the upregulation of *HK3* gene in CRC and confirmed its involvement in tumorigenesis. Expression of *HK3* gene significantly correlated with mRNA levels of important EMT transcriptional factors (*ZEB2, TGFB3, TGFB1, SNAI2,* and *TWIST1*) and components (*GREM1* and *COL1A1*). Thus, our study suggests that HK3 may participate in EMT process and that its upregulation could be one of the crucial changes for adaptation of glucose metabolism to EMT in CRC.

## Additional files

Additional file 1: Table S1. (XLS 393 kb)

Additional file 2: Table S2. (XLS 389 kb)

Additional file 3: Table S3. (XLS 34 kb)
